# Partial night lighting may reduce the physiological impact of artificial light at night on captive zebra finches

**DOI:** 10.3389/fphys.2025.1592407

**Published:** 2025-05-30

**Authors:** Rachel R. Reid, Neal Dawson, Eleanor Duncan, Robert Gillespie, Christopher Mitchell, Claire J. Branston, Pablo Capilla-Lasheras, Jelle Boonekamp, Davide M. Dominoni

**Affiliations:** ^1^ School of Biodiversity, One Health and Veterinary Medicine, Graham Kerr Building, University of Glasgow, Glasgow, United Kingdom; ^2^ Environment and Sustainability Institute, University of Exeter, Cornwall, United Kingdom; ^3^ School of Health and Life Sciences, University of the West of Scotland, Glasgow, United Kingdom; ^4^ Swiss Ornithological Institute (Vogelwarte), Sempach, Switzerland; ^5^ Doñana Biological Station, Spanish National Research Council (EBD-CSIC), Sevilla, Spain

**Keywords:** avian health, avian physiology, artificial light, light pollution, urban ecology

## Abstract

**Background:**

Artificial light at night (ALAN) continues to increase at an unprecedented rate globally every year. ALAN can disrupt circadian rhythms and cause behavioural and physiological changes which may have knock on effects for health, yet we still understand very little about these effects. It is becoming increasingly important to investigate potential mitigation strategies, through understanding what aspects of ALAN negatively impact wildlife health.

**Methods:**

Here we present the results of an experiment where we investigated the impact of ALAN on various health biomarkers in 44 captive adult zebra finches *(Taeniopygia guttata)* over the course of 4 months. The health biomarkers measured included glucose concentration, change in relative telomere length, malondialdehyde, and antioxidant capacity of plasma. The birds were separated into three treatment groups consisting of 13–16 individuals and were either exposed to full light at night, partial light at night or darkness (control).

**Results:**

We show that exposure to full light at night impacted the circadian pattern of glucose levels, with glucose concentrations remaining elevated later into the night compared to the control group. Full light at night also accelerated telomere shortening. However, the relationship between telomere length and treatment only became significant when the partial light at night treatment group was removed and should therefore be interpreted with caution. These effects were not observed under partial light at night.

**Conclusion:**

Our findings suggest that partial night lighting may mitigate some of ALAN’s negative impacts on wildlife health. This approach could be a valuable tool in future strategies to reduce the ecological effects of light pollution in urban environments and should be investigated further.

## 1 Introduction

As our world has become increasingly urbanised due to continued human population growth, the levels of various novel stressors associated with urban environments have increased. One such stressor is artificial light at night (ALAN). It is estimated that over 80% of the human population globally now lives with light pollution (Guan et al., 2022). ALAN can be attributed to various sources including street lighting, architectural lighting, security lighting, domestic lighting and vehicle lighting (Gaston et al., 2013). It also contributes to skyglow where ALAN that is emitted or reflected upwards is scattered by molecules in the atmosphere (Gaston et al., 2013). Recent estimates indicate that observable light emissions are increasing faster than the human population globally (Gaston and Sánchez De Miguel, 2022). While ALAN provides social and economic benefits for humans (Gaston and Sánchez De Miguel, 2022), the loss of natural darkness may have significant negative consequences, particularly for wildlife.

There are both direct and indirect ways in which exposure to ALAN can negatively impact wildlife. The earth’s rotation produces natural daily dark-light cycles that regulate an organism’s behaviour and physiology (Russart K. and Nelson R., 2018). Most organisms have evolved circadian clocks that align internal biological processes with external environmental cues (Kumar, 1997; Kumar et al., 2004; Yadav et al., 2022). This synchronisation optimises the timing of behaviours such as feeding, resting, reproduction, and migration, ultimately enhancing survival and reproductive success (Van Doren et al., 2017; Aulsebrook et al., 2018; Hoffmann et al., 2018; Russart K. L. G. and Nelson R. J., 2018). Several studies have shown that many diurnal species are active throughout the night when exposed to ALAN, expending more energy than needed and diverting resources away from important physiological functions (Dominoni et al., 2013; De Jong et al., 2017; Dimovski and Robert, 2018; Yadav et al., 2022). In addition to behaviour, many physiological processes are under circadian control and are therefore vulnerable to ALAN exposure (Kumar, 1997; Kumar et al., 2004). These physiological processes include, but are not limited to, the secretion of corticosterone (Helm et al., 2024) and sex steroids (Helm et al., 2024), and the production of glucose and insulin (Lobban et al., 2010; Kumar Jha et al., 2015). The mismatch between internal circadian timing and the external environment is especially problematic during energetically demanding periods such as winter (Jansen et al., 2016; Sletten et al., 2022) or the breeding season (Helm and Visser, 2010; Williams et al., 2017; Walker et al., 2019), when efficient energy management is crucial for survival and fitness.

The misalignment of internal body rhythms with the external environment is known to negatively impact human health, including increasing the risk of metabolic syndromes, obesity, as well as breast and prostate cancer (Erdem et al., 2017; Russart K. and Nelson R., 2018). However, the health impacts of ALAN on wildlife have only recently been appreciated. In both nocturnal and diurnal rodents, ALAN reduces survival rates and reproductive success (Vardi-Naim et al., 2022). ALAN has been shown to reduce reproductive success and impair both larval development and pupal diapause in moth species (Boyes et al., 2021). There is also evidence of a negative relationship between ALAN and health in bird species. In male great tits *(Parus major)*, circadian disruption altered tissue function related to timing, memory, metabolism and immunity (Dominoni et al., 2022). Another study showed exposure to white light led to increased nighttime activity and reduced oxalic acid levels in great tits, indicating sleep deprivation which correlated with a higher likelihood of avian malaria infection (Ouyang et al., 2017).

Although many studies show negative impacts of ALAN on health, others report minimal or no effects. For example, ALAN had little effect on the fitness and reproductive success of two songbird species (De Jong et al., 2015). Similarly, in great tits, no difference was found in telomere length between birds exposed to white light and the control group, despite a higher prevalence of malaria infection (Ouyang et al., 2017). It has also been shown that there was no direct evidence for increased oxidative stress levels in tammar wallabies (*Notamacropus eugenii)* exposed to ALAN (Dimovski and Robert, 2018). These contrasting findings suggest that the impact of ALAN on health is complex and may vary depending on factors such as species, biomarkers measured, life stage, study duration, and environment, as has also been observed for urbanisation-related health effects on wildlife (Reid et al., 2024).

To understand health and physiology, many studies rely on a biomarker-based approach. This is because physiological responses play a critical role in helping organisms cope with stress, and many physiological processes have a suite of measurable components that can serve as biomarkers (Hagger et al., 2006; Isaksson, 2020). However, this approach comes with limitations. Most studies investigating the effects of ALAN tend to focus on one or two biomarkers of health (Evans et al., 2012; Raap et al., 2017; Dominoni et al., 2018; Malek and Haim, 2019). Yet, health is a multivariate and multidimensional trait, shaped by complex interactions between physiological systems (Isaksson, 2020). Relying on a narrow set of biomarkers produces the risk of missing key effects and interactions. By measuring a broader panel of biomarkers, researchers can gain a more holistic understanding of health allowing for better detection of trade-offs and interactions among systems. Several biomarkers are particularly relevant in the context of ALAN, as many physiological processes are under circadian regulation. For example, circadian synchronisation is thought to be important in reducing the risk of pathologies such as cancer and premature ageing (Krishnan et al., 2008).

In many vertebrate species, glucose levels are tightly regulated within a narrow range to maintain energy homeostasis, but this regulation is sensitive to ecological and physiological factors such as habitat quality, reproductive state, and body condition (Polakof et al., 2011; Giridharan, 2018). Glucose also shows daily rhythmicity, making it vulnerable to disruption by ALAN (Lobban et al., 2010; Kaliński et al., 2014; Kumar Jha et al., 2015). Circadian misalignment can impair energy homeostasis, contributing to conditions such as diabetes and obesity, and increasing the risk of hypoglycaemia or hyperglycaemia in birds (Braun and Sweazea, 2008; Kawahito et al., 2009; Kumar Jha et al., 2015; Sojka, 2025). However, it is also important to recognise that birds naturally maintain higher blood glucose levels than mammals of comparable body size (Sweazea, 2022). These elevated levels are not pathological but adaptive, supporting high energy activities such as long-distance migration and potentially increasing survival probability (Montoya et al., 2022; Sojka, 2025; Hawkes, 2025).

Telomere attrition is another biomarker that may be affected by ALAN, as telomerase activity exhibits endogenous circadian rhythms (Chen et al., 2014; Ledda et al., 2020). Telomere attrition is linked to fitness-related traits such as survival, lifespan, and reproductive success, (Beirne et al., 2014; Wilbourn et al., 2018; Wood and Young, 2019), and environmental stressors are known to accelerate telomere loss (Monaghan and Haussmann, 2006). Similarly, several components of oxidative stress are under circadian regulation which allows for the maintenance of redox balance (Hardeland et al., 2003; Lai et al., 2012; Budkowska et al., 2022). Oxidative stress is linked to many important processes including senescence, ornamentation, sperm performance, survival, and reproduction (Sepp et al., 2012; Speakman et al., 2015). To properly interpret oxidative stress, it is recommended that researchers should measure both oxidative damage markers and antioxidant defences (Costantini and Verhulst, 2009; Speakman et al., 2015; Costantini, 2019), reinforcing the importance of a multi-biomarker approach.

Given the growing evidence of ALAN’s impact on wildlife health and biodiversity (Sanders et al., 2021), developing mitigation strategies is becoming increasingly important. Adjusting ALAN properties, such as intensity and spectrum, is a promising avenue. Studies indicate that reducing light intensity (Evans et al., 2012; Raap et al., 2017; Dominoni et al., 2018) and shifting the light spectrum to longer wavelengths (De Jong et al., 2017; Aulsebrook et al., 2020) can significantly lessen ALAN’s behavioural and physiological impacts. Another promising approach is partial night lighting, which involves reducing the duration of nighttime lighting exposure, typically by switching off lights after midnight, when human activity is reduced. This strategy aims to balance ecological protection with human safety and energy efficiency (Gallaway et al., 2010; Gaston et al., 2012; 2015; Pagden et al., 2020). Partial night lighting is already implemented in parts of the UK and other regions, yet its efficacy as a mitigation strategy against the negative impacts of ALAN remains understudied. Studies that have looked into partial night lighting have found mixed results. One study found stronger negative effects on aphid colony growth under partial light compared to exposure to full light at night (Heinen et al., 2023). Studies on bats reported no benefits of partial light on bat activity and suggested to be beneficial the lights would need to be switched off before midnight (Azam et al., 2015; Day et al., 2015). However, in contrast, partial lighting reduced disruptions to oyster behavioural rhythms (Botté et al., 2023). Therefore, there is mixed evidence to date for the benefits of partial night lighting as a mitigation strategy. This highlights the need for further experiments to determine whether partial ALAN exposure has weaker health impacts than full ALAN exposure. Moreover, most experimental studies, particularly in captivity, involve short-term ALAN exposure (days to weeks). However, in one long-term study, Eurasian blackbirds *(Turdus merula)* exposed to ALAN for 2 years experienced a complete reproductive shutdown after the first year (Dominoni et al., 2013). This highlights the potential for dramatic long-term effects of ALAN and the need for longitudinal studies. (Alaasam et al., 2024; Beaugeard et al., 2024; Dimovski and Robert, 2018; Itay and Haim, 2024; Jha and Kumar, 2017; Malek et al., 2019).

In this study, we exposed adult zebra finches (*Taeniopygia guttata)* to ALAN for 4 months in captivity to examine effects on health over time. Zebra finches are widely used in ecological studies, particularly in the fields of physiology and neurology. As a species, they are easy to keep and breed in captivity, providing opportunities for controlled observations that would not be possible in wild systems (Griffith and Buchanan, 2010; Mak et al., 2015). Birds were divided into three groups: A natural photoperiod without ALAN (hereafter DARK), ALAN throughout the night (FLAN), and ALAN during the first half of the night (PLAN). The PLAN and FLAN groups were exposed to the same intensity of ALAN. The PLAN group was designed to expose the birds to light during the first half of the night to test the effects of intermittent darkness on health. The timing used in the treatment groups was aligned with the winter photoperiod at the time of the experiment. To capture the multivariate nature of health, we measured several biomarkers including glucose concentration, relative telomere length (RTL), antioxidant capacity of plasma (OXY) which is a measure of antioxidant defence and malondialdehyde (MDA) which is a product of lipid peroxidation.

To investigate the physiological consequences of ALAN, we tested the hypothesis that ALAN disrupts important biological processes over time. Specifically, we predict that ALAN alters the circadian rhythm of glucose, leading to abnormal peaks in glucose concentrations, particularly in the FLAN group, with a reduced effect in the PLAN group. We further hypothesis that ALAN exposure will increase overall glucose levels over time, potentially due to disrupted circadian regulation causing changes in activity and feeding patterns (Kumar Jha et al., 2015; Rumanova et al., 2022), with a stronger effect expected in the FLAN group. In addition, we predict that ALAN will accelerate telomere shortening over time, and that this effect will be more pronounced under FLAN conditions. Lastly, we predict that ALAN will elevate oxidative stress over time, evidenced by increased MDA concentrations and decreased OXY, again with a stronger impact in the FLAN group.

## 2 Methods

### 2.1 Ethics statement

The experiment was approved by the Home Office under the project licence P6859F36E to D.M.D. All blood samples were taken by Personal licence holders.

### 2.2 Experimental protocol

On the seventh of September 2022, 44 adult (>3 months old) zebra finches (21 females and 23 males (Barkan et al., 2007) were brought to a facility based at the University of Glasgow having been bred together in captivity from the same breeding stock. The birds were given a 2-week period of acclimatisation where they were exposed to a photoperiod of 9 h daylight and 15 h of darkness (lights on from 7 a.m. to 4 p.m.) which aligned with the local photoperiod. Between the 21st of September 2022 and the 23rd of January 2023, the birds were split into and kept in three treatment groups which varied in their lighting regimes, these are described in Table 1 and Supplementary Figure S1A. The males and females were housed separately, and individuals were randomly selected for each treatment group as shown in Supplementary Figure S1B.

**TABLE 1:** Code names, description and time of light exposure for each of the treatment groups that will be referred to throughout the main text. The associated sample sizes, in number of individuals, are also shown.

Code	Treatment	Time lights were on	Sample size
Full darkness at night (DARK)	Control group – lights switched off during the night	Lights on from 7 a.m. to 4 p.m. and then 15 h of darkness	13
Partial light at night (PLAN)	Group exposed to partial artificial light at night during the dark photoperiod	7 a.m. to 4 p.m. and 9 p.m. to 2 a.m. therefore experienced 10 h of darkness	15
Full light at night (FLAN)	Group exposed to dim light at night throughout the dark photoperiod	9 a.m. to 4 p.m. and 7 p.m. to 9 a.m. therefore experienced 3 h of darkness	16

Each treatment took place in a separate room to prevent cross-exposure between conditions. Each treatment room had a layout of four cages (2 m × 1 m × 0.5 m), housing 2–5 birds per cage (Supplementary Figure S1B). Each cage was fitted with two broad spectrum white LED lights (Broadcom ASMT-UWB1-NX3E2) that covered a wavelength of 400–700 nm and were standardised to produce an intensity of 1.5 lux at perch level. Each diode produces a luminous intensity of 2.33 candela at 20 mA forward current, with a broad viewing angle of 120°, ensuring lateral light dispersion across the environment. Lights were controlled *via* a mechanical timer. The daytime illumination was produced by overhead ceiling lights and produced an intensity of 400 lux at perch level. Birds were provided with dry finch seed mix and water *ad libitum*.

### 2.3 Sample collection

We blood sampled each bird at the beginning (21 September 2022) and end (23 January 2023) of the experiment. We took blood samples from the brachial vein with a sterile needle into a 75 µL capillary tube, taking no more than 1% of each individual’s body weight. We immediately dispensed the blood into a prelabelled 1.5 mL Eppendorf tube, the samples were kept on ice until processed. Within 4 hours of collection, we spun the samples using a centrifuge at 2,000 rpm at 4^o^c for 10 min to separate the plasma from the red blood cells (RBC’s) and then transferred the samples into a −80°C freezer. During each sampling attempt we weighed every bird using an electronic balance.

To ensure that we could investigate the effect of treatment on the circadian rhythm of glucose, at the end of the experiment we took blood samples from each bird twice at different times of day which included 1 a.m., 6 a.m., 1 p.m. and 8 p.m. These times were chosen to ensure we could obtain measurements over a 24-h period. Half of the birds were sampled at each timepoint to ensure each bird was only sampled twice and had a break (12–14 h) in between sampling efforts. This staggered sampling approach meant that there were birds from every treatment group within each sampling time. To minimize handling stress, blood samples were collected as quickly as possible following capture, with a mean bleed time of 3.1 ± 1.5 (range: 1–7 min).

### 2.4 Glucose measurements

During the start and end of experiment sampling attempts, we used a drop of blood from each individual bird used to measure Glucose levels using the Exactive EQ real-time blood glucose test (MicroTech Medical, Zhejiang, China).

### 2.5 Measuring relative telomere length

We extracted DNA from RBC’s using the Puregene kit (QIAGEN, Hilden, Germany) following the manufacturers protocol. We quantified the concentration and quality of DNA using a Nanodrop-8000 spectrophotometer (Thermoscientific, Waltham, Massachusetts, United States), we then diluted the samples to 1.25 ng/μL and stored at −80°C.

We then measured Relative Telomere Length (RTL) using real-time quantitative polymerase chain reaction (qPCR) which has been validated by various studies (Cawthon, 2002; Criscuolo et al., 2009). RTL was measured as the T/S ratio (telomere repeat copy number (T) to control gene copy number (S) relative to a reference sample). The control gene, *RAG-1* (Groth and Barrowclough, 1999; Liu et al., 2018), was chosen for being highly conserved across species, including zebra finches (Kumar et al., 2015). Telomere primers were Tel1b 5′CGGTTTGTTTGGGTTTGGGTTTGGGTTTGGGTTTGGGTT-3′ and Tel2b 5′-GGCTTGCCTTACCCTTACCCTTACCCTTACCCTTACCCT-3′. *RAG-1* primers were 5′-GCAGATGAACTGGAGGCTATAA-3′ and 5′-CAGCTGAGAAACGTGTTGATTC-3′.

We performed primer optimisation tests with concentrations of 50, 100, 300, 500 nM. We conducted the qPCR assays with 7.5 ng of DNA per reaction with a primer concentration of 50 nM (*RAG-1*) and 500 nM (Telomere) in a final reaction volume of 20 µL containing 10 µL (1x) of absolute blue qPCR SYBR green low ROX mix. Thermal profiles were as follows: Telomere assay - 15 min at 95°C, 27 cycles of 15s at 95°C, 30s at 58°C, and 30s at 72°C; *RAG-1* assay - 15 min at 95°C, 40 cycles of 15s at 95°C and 30s at 60°C. We then performed melting curve analysis after each reaction. All reactions were run in triplicate, and plates were balanced for cage number and treatment group to minimise experimental bias. We ran a standard curve (6 points, 40–1.25 ng) on each plate using a serial dilution of high-quality pooled DNA samples extracted from several individuals in the study. We used 6 µL of on each plate as non-template controls. We also included two inter-plate control samples on each plate by mixing two separately selected batches of samples, using 7.5 ng of DNA per reaction. We then used these control samples to calculate inter-plate variation.

We analysed the qPCR data using QBASE software (Hellemans et al., 2008) which calculates RTL (T/S ratio) as calibrated normalized relative quantities, controlling for amplification efficiency and inter-run variation by including three inter-run calibrators from the standard curve. The mean telomere assay efficiency was 99.2% ± 2.3% and 104.1% ± 1.9% for the *RAG-1* assays. Mean intra- and inter-plate of C_t_ values were 2.14% and 2.97% for telomere reactions and 1.02% and 1.10% for the RAG-1 reactions.

### 2.6 Measuring malondialdehyde (MDA) levels in plasma

We quantified plasma concentrations of MDA using high-performance liquid chromatography (HPLC) following Nussey et al., 2009; Nussey et al., 2009). In summary, we transferred an aliquot of plasma to a reaction tube pre-loaded with 20 µL of butylated hydroxytoluene (BHT). We then added 40 µL of thiobarbituric acid (TBA) and 160 µL of phosphoric acid. Next, we vortexed reaction tubes for 5 s and placed them in a dry heat bath at 100°C for 1 h, before being cooled on ice. Once the reaction tubes had cooled, we added 160 µL of butanol and then vortexed the samples for 10 s, we then centrifuged the samples at 4°C at 12,000 × g for 3 min. An aliquot (60 µL) of the upper phase which contained MDA-TBA adducts was drawn off and placed in a HPLC vial.

We then injected 20 µL of the samples into a HPLC system (Agilent 1200 series) fitted with a Hewlett-Packard Hypersil 5 µL ODS 100 × 4.6 mm column and a 5 µL ODC guard column (Thermo Fisher Scientific Inc., Massachusetts, United States) kept at 37°C. The mobile phase was MeOH/buffer (40:60 v/v) at a flow rate of 1 mL min^−1^. The buffer was 50 mM potassium monobasic phosphate which we adjusted to pH 6.8 using 5M KOH. We then performed Fluorescence detection using an Agilent 1260 at 515 nm excitation and 553 emission. We calibrated the MDA concentration’s using an external standard of 1,1,3,3- tetrathoxypropane (TEP) serially diluted with 40% ethanol. We calculated the intra-plate coefficient of variation (CV) for this assay using 12 standards run in duplicate for the calibration curve, yielding an intra-plate CV of 4.16%. Due to limited sample availability, we could not directly assess repeatability between plates. However, this assay has been demonstrated to be repeatable in previous analyses conducted in the same laboratory (Nussey et al., 2009; Bodey et al., 2020).

### 2.7 Measuring antioxidant capacity of plasma

We measured antioxidant capacity of plasma (OXY) using an OXY-Adsorbent test (Diacron International, Grosseto, Italy) following the manufacturer’s instructions with modifications. We ran all samples in triplicate. We first diluted 2 µL of plasma to 1:100 and then we added 5 µL of the diluted sample to the plate along with 190 µL hypochlorous acid (HCIO; an endogenously produced oxidant). Once the oxidant solution was added, we incubated the plate at 37°C for 10 min. After incubation we added 5 µL of chromogen (alkyl-substituted aromatic amine) to each of the wells. We read the plate using SpectraMax plus spectrophotometer (Molecular devices, San Jose, California, United States) at a temperature of 37°C and a wavelength of 505 nm. The results of the OXY-Adsorbent test were expressed as µmol of HCI0/ml of sample. We calculated repeatability by repeating every step of the protocol for 20 samples, we ran a linear mixed effects model (LMM) using sample ID as a random effect to account for repeated measures within individuals. Repeatability was then calculated as the ratio of the random intercept variance to the total variance.

The between plate repeatability was R^2^ = 0.57 (N = 20). The intra-plate CV calculated using these same 20 samples was 9.35% and the inter-plate CV was 10.97%.

### 2.8 Statistical analysis

We performed all analysis in R (version 4.0.4) (R core team 2024) using the “lme4” (Bates et al., 2015) package. We conducted model selection *via* backward selection using likelihood-ratio tests (LRTs) with the “drop1” function. We removed non-significant interactive terms from the initial full models however we retained all noninteractive fixed effect predictors in the full model even if not significant. We performed *post hoc* analysis using the Tukey test for significant interactions using the “Emmeans” package (Lenth et al., 2024). We included a unique ID for each bird as a random effect in all models. As the number of birds kept in each cage varied, we included group size as an explanatory variable in every model. We then checked residuals for normality using a quantile-quantile plot. All models were run using the “lmer” function.

To investigate the impact of treatment on glucose circadian variation over time, we ran a linear mixed model (LMM) with time of day, treatment, sex, body mass and a three-way interaction with time of day, sex and treatment as explanatory variables. This interaction tested whether treatment influenced glucose variation over time and if this effect was sex dependent.

We then analysed glucose levels over time with an LMM, using glucose level as the response variable. Experiment timepoint (Start, End), body mass, sex, treatment, and a three-way interaction with experiment timepoint, sex and treatment were included as explanatory variables. We subset the data to include samples from midday (1 p.m.) and early evening (8 p.m.) matching sampling times at the start of the experiment, to account for the circadian pattern in glucose levels.

To investigate effects of treatment group on changes in RTL, MDA and OXY between the start and end of the experiment, we ran LMMs for each metric, with sex, treatment, experiment timepoint, body mass and a three-way interaction between sex, treatment and timepoint as explanatory variables. We included plate ID as a random effect where appropriate. In all models, we also tested any associated two-way interactions as well as the three-way interactions and compared model fit using LRTs.

## 3 Results

### 3.1 The impact of ALAN on the circadian rhythm of glucose

**FIGURE 1:**
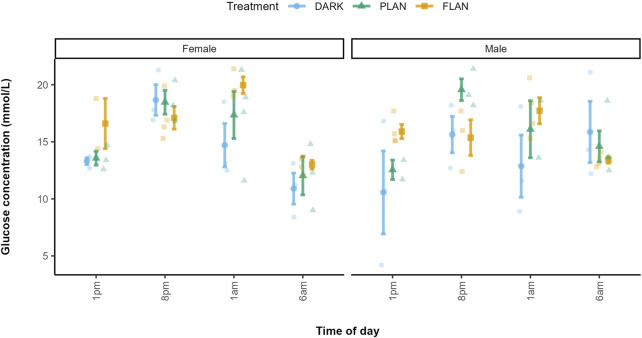
The relationship between treatment group and glucose levels in zebra finches at various times of day. The DARK group are shown in blue, the PLAN group are shown in green, and the FLAN group are shown in yellow. The means are shown on the plot along with the standard errors. The raw data points can also be seen. Time of day is shown on the x-axis and glucose concentration is shown on the y-axis. The first panel of the plot represents the females, and the second panel represents the males. Sample sizes are as follows DARK Female 1 p.m. (3), DARK Male 1 p.m. (3), PLAN Female 1 p.m. (3), PLAN Male 1 p.m. (2), FLAN Female 1 p.m. (2), FLAN Male 1 p.m. (4), DARK Female 8 p.m. (3), DARK Male 8 p.m. (3), PLAN Female 8 p.m. (3), PLAN Male 8 p.m. (3), FLAN Female 8 p.m. (4), FLAN Male 8 p.m. (3), DARK Female 1 a.m. (3), DARK Male 1 a.m. (3), PLAN Female 1 a.m. (2), PLAN Male 1am (4), FLAN Female 1 a.m. (3), FLAN Male 1 a.m. (4), DARK Female 6 a.m. (3), DARK Male 6 a.m. (3), PLAN Female 6 a.m. (3), PLAN Male 6 a.m. (4), FLAN Female 6 a.m. (4), FLAN Male 6 a.m. (4). Tukey’s *post hoc* analysis revealed significant and marginally significant differences between the FLAN and DARK groups in males at 1 p.m. (P = 0.042) and at 1 a.m. (P = 0.062), as well as in females at 1 a.m. (P = 0.070).

**FIGURE 2:**
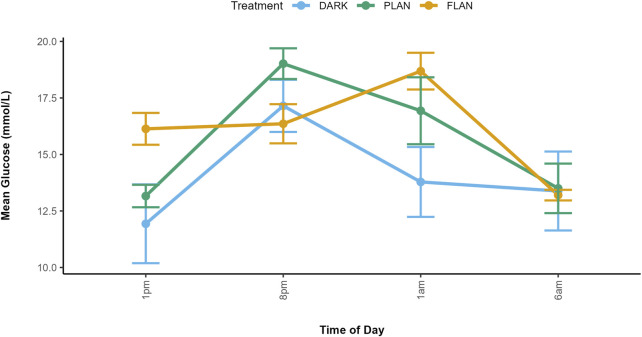
Mean glucose concentrations along with standard errors are shown at four time points for each treatment group. Glucose concentration is shown on the y-axis and time of day is shown on the x-axis. The DARK group are shown in blue, the PLAN group are shown in green, and the FLAN group are shown in orange. Samples size are as follows: DARK (12), PLAN (15), FLAN (16).

Our analysis revealed a significant three-way interaction between sex, treatment and time of day on glucose levels (*X* ^2^ = 31.90, df = 17, P = 0.015 Figure 1; Supplementary Table S1). At 1 p.m., the FLAN group had higher glucose levels than the PLAN and DARK groups in both males and females. At 1 a.m. females in the FLAN group had higher glucose levels than both the PLAN and DARK groups, PLAN also had higher glucose levels than the DARK group. This also was the case for males, though less pronounced. Post hoc analysis revealed that males in the FLAN group had significantly higher glucose levels than the DARK group at 1 p.m. [mean estimate (95% Confidence Interval, CI) = −6.535 (−12.85, −0.224), P = 0.041, Figure 1; Supplementary Table S2]. For females’ glucose levels in the FLAN group were significantly higher than in the DARK group at 1 a.m. [mean estimate (95% Confidence Interval, CI) = −5.364 (-11.08, 0.347), P = 0.07, Figure 1; Supplementary Table S2]. No significant pairwise differences were found between DARK and PLAN or FLAN and PLAN (Supplementary Table S2). The PLAN and DARK groups appeared to follow the same general pattern over time however birds exposed to PLAN had higher glucose levels than the DARK group at 8 p.m. and 1 p.m. (Figure 2). The birds exposed to FLAN had much higher glucose levels than the other groups at 1 p.m. and 8 p.m., this then increased at 1 a.m. and did not decrease until 6 a.m. (Figure 2).

In the DARK (females) and PLAN (both sexes) groups glucose levels peaked at 8 p.m., however in the FLAN group for both sexes peaked at 1 a.m. (Figure 1).

### 3.2 The impacts of ALAN on glucose concentrations over the course of the experiment

A trend for a three-way interaction between treatment, sex, and experiment timepoint was observed when comparing glucose levels at the start and end of the experiment (*X* ^2^ = 12.97, df = 7, P = 0.073), however there was no effect of any of these factors individually (Supplementary Table S3). Given the trend towards a three-way interaction between treatment, sex, and experiment timepoint in the full model, we conducted follow-up exploratory analysis to further investigate whether this interaction was being driven by specific treatment groups. To do this, we modelled the PLAN and FLAN groups separately against the DARK group. This allowed us to isolate and interpret the directionality of interaction effects that may have been obscured in the full model. No significant effect of treatment on glucose levels was found between the DARK and PLAN groups (*X* ^2^ = 0.42, df = 1, P = 0.519). However, a significant three-way interaction between sex, experiment timepoint, and treatment was found when comparing the DARK and FLAN groups (*X* ^2^ = 10.66, df = 4, P = 0.031).

**FIGURE 3:**
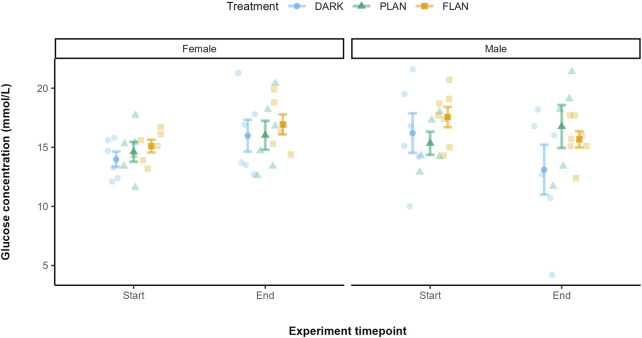
The relationship between treatment groups and glucose levels in zebra finches from the start to the end of the experiment. The DARK group are shown in blue, the PLAN group are shown in green, and the FLAN group are shown in yellow. The panels represent the different sexes. The means are shown along with the standard errors, the raw data points are also shown. Experiment timepoint is shown on the x-axis and glucose concentration is shown on the y-axis. Samples sizes are as follows DARK Female (6), DARK Male (6), PLAN Female (6), PLAN Male (5), FLAN Female (6), FLAN Male (7). Tukey’s *post hoc* test revealed significant differences (when PLAN was removed from the model) between males and females in the DARK group at the end of the experiment (P = 0.049).

Figure 3 shows that, in females, glucose levels increased over the course of the experiment in all treatment groups, with the FLAN group having the highest levels by the end. In males, the FLAN group had higher glucose levels at the start of the experiment than both PLAN and DARK. The males in the DARK group showed a decrease in glucose over time, with FLAN males experiencing a less pronounced decline. Post hoc analysis comparing the FLAN and DARK treatment groups showed a significant difference in glucose levels between the start and end of the experiment in DARK males [mean estimate (95% Confidence Interval, CI) = 3.26 (0.403, 6.12), P = 0.027]. However, the effect size for this contrast was small (Cohen’s d = 1.38) suggesting limited practical significance. Additionally, there was a significant difference between males and females in the DARK group at the end of the experiment [mean estimate (95% Confidence Interval, CI) = 3.78 (0.010, 7.54), P = 0.049], with a small effect size (Cohen’s d = 1.62).

We also tested if glucose levels over the course of the experiment were impacted by MDA levels and if there differed under the different treatment groups. We found a significant effect of MDA on glucose levels (*X* ^2^ = 4.328, df = 1, P = 0.037), however this was not dependent on treatment or experiment timepoint, suggesting that MDA may influence glucose independently of ALAN exposure.

### 3.3 The impacts of ALAN on telomere length over time

**FIGURE 4:**
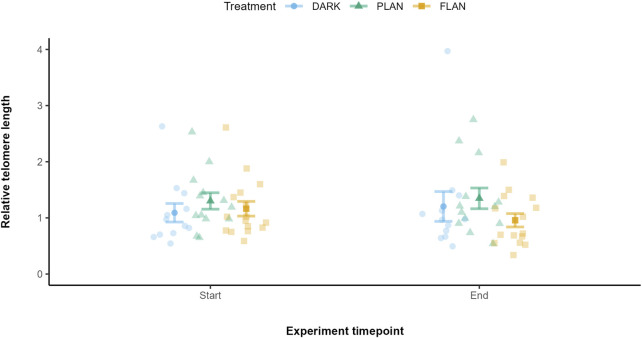
The relationship between treatment groups and telomere length in zebra finches at the start and end of the experiment. The DARK group are shown in blue, the PLAN group are shown in green, and the FLAN group are shown in yellow. The model residuals (without any plate effects) corrected by mean telomere length along with the standard errors, the raw data points are also shown. Experiment timepoint is shown on the x-axis and relative telomere length is shown on the y-axis. Sample sizes are DARK (12), PLAN (15), FLAN (16). LRT analysis revealed a significant interaction between treatment and experimental timepoint when the PLAN group was removed from the full model (P = 0.016).

We first included all three treatment groups in the same model to look at the effect on telomere length over time. There was a trend between treatment and experiment timepoint on telomere length however this was not significant (*X* ^2^ = 4.81, df = 2, P = 0.090). To explore this further, we modelled PLAN and FLAN separately with the DARK group. When comparing the DARK and PLAN groups there was no significant effect of treatment on telomere length (*X* ^2^ = 0.407, df = 1, P = 0.524, Figure 3). However, when comparing the DARK and FLAN treatment groups there was a significant interaction between treatment and experiment timepoint (*X* ^2^ = 5.80, df = 1, P = 0.016, Figure 3). However, as this trend was not supported in the full model, this result should be interpreted with caution. Telomere length declined from the start to the end of the experiment in the FLAN group, whereas this was not observed in the DARK or PLAN groups as shown in Figure 4. The final model output is shown in Supplementary Table S4.

We also tested if there was an effect of average MDA levels per individuals on telomere loss, but we found no significant effect (*X* ^2^ = 0.12, df = 1, P = 0.732, Supplementary Figure S2). All treatment groups were shown to overlap in their telomere shortening over time (Supplementary Figure S2).

### 3.4 The impact of ALAN on MDA levels over time

**FIGURE 5:**
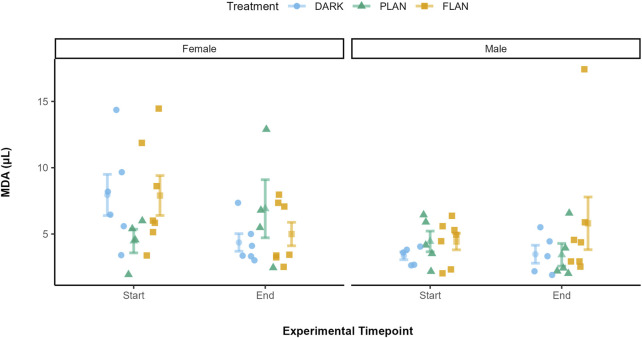
The relationship between treatment groups and MDA in zebra finches at the start and end of the experiment. The DARK group are shown in blue, the PLAN group are shown in green, and the FLAN group are shown in yellow. The means are shown along with the standard errors, the raw data points are also shown. Experiment timepoint is shown on the x-axis and MDA is shown on the y-axis. The separate panels represent the different sexes. Sample sizes are as follows are DARK Female (6), DARK Male (5), PLAN Female (4), PLAN Male (5), FLAN Female (7), FLAN Male (7). LRT analysis revealed no significant effect of treatment on MDA levels although there was a significant effect of sex (P = 0.019).

There was no significant effect of any of the interactions tested on MDA levels. Treatment did not have a significant impact on MDA levels (*X* ^2^ = 0.23, df = 2, P = 0.891, Supplementary Table S5). There was also a significant effect of sex on MDA levels (*X* ^2^ = 5.49, df = 1, P = 0.019, Supplementary Table S5). Figure 5 shows that, when comparing the treatment groups for females, MDA levels increased from the start to the end of the experiment only in the PLAN group while levels decreased in the FLAN and DARK groups. However, when we consider the males their MDA levels only increased in the FLAN group.

### 3.5 The impact of ALAN on OXY levels over time

**FIGURE 6:**
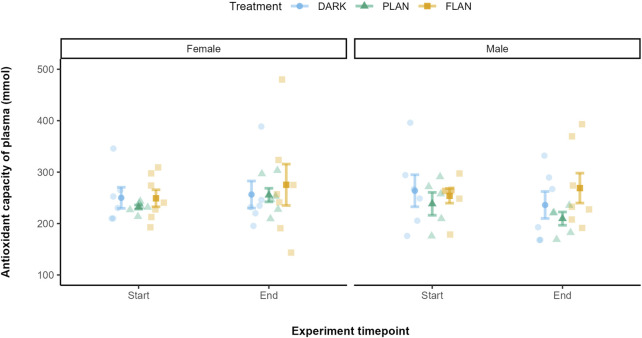
The relationship between treatment groups and OXY in zebra finches at the start and end of the experiment. The DARK group are shown in blue, the PLAN group are shown in green, and the FLAN group are shown in yellow. The model residuals (without any plate effects) corrected for the mean OXY levels are shown the standard errors, the raw data points are also shown. Experiment timepoint is shown on the x-axis and OXY is shown on the y-axis. The separate panels represent the different sexes. Sample sizes are as follows DARK Female (6), DARK Male (6), PLAN Female (6), PLAN Male (5), FLAN Female (7), FLAN Male (7). No significant treatment effects were detected.

There was no significant effect of any of the interactions tested on OXY levels. There was no significant effect of treatment (*X* ^2^ = 1.08, df = 2, P = 0.583, Supplementary Table S6) on OXY levels. In both the DARK and PLAN groups OXY levels did not appear to change by much from the start to the end of the experiment (Figure 6). The birds exposed to FLAN appeared to have an increase in their OXY levels by the end of the experiment, but this was not significant.

We also tested whether MDA had an effect of OXY over the course of the experiment and if this differed in the different treatment groups. We found no significant relationship between MDA and OXY (*X* ^2^ = 0.007, df = 1, P = 0.931).

## 4 Discussion

It is well documented in the literature that organisms that are exposed to ALAN face both behavioural and physiological changes that can be detrimental to health. The results of this study show that, when exposed to full light at night birds are potentially experiencing circadian misalignment of glucose levels, where they are likely experiencing altered feeding behaviour and changes to their activity-rest cycles. Birds exposed to FLAN also experienced accelerated telomere attrition. Interestingly, these effects of ALAN were not present when birds were exposed to PLAN. Neither ALAN treatment group had an effect on oxidative stress levels. These results could have important implications for the management of lighting regimes, as they suggest that reducing the duration of outdoor illumination may reduce negative physiological impacts on songbirds. Future studies would apply this to other wildlife species.

### 4.1 The effect of ALAN on glucose levels

In this study, we used two complementary approaches to assess the effects of ALAN on glucose regulation. First, to investigate circadian variation, we measured blood glucose at four time points within a single 24-h period. This allowed us to assess how glucose concentrations fluctuated throughout the day and night under different lighting treatments. Second, to assess long-term effects of ALAN on glucose regulation, we measured glucose at the start and end of the 4-month experimental period, at approximately the same time of day for each individual.

Glucose levels have been shown to be under strong circadian control in several species (Remage-Healey and Romero, 2000; Lobban et al., 2010; Kalsbeek et al., 2014; Kumar Jha et al., 2015). Glucose levels typically peak during active periods or when the organism is eating and are stored during rest phases to meet the metabolic demands throughout the day (Remage-Healey and Romero, 2000; Downs et al., 2010; Lobban et al., 2010). Our results clearly indicated that glucose levels in captive zebra finches varied throughout the day. Exposure to FLAN caused glucose levels to peak at 1 a.m., in contrast to the PLAN and DARK groups, where peaks occurred at 8 p.m., followed by a decline. Therefore, exposure to PLAN did not appear to be enough to disrupt the circadian pattern of glucose. However, no significant pairwise difference was observed between the FLAN and PLAN groups at any of the sampling times, possibly due to low statistical power caused by a small sample size. There are limited studies that have investigated the effects of ALAN exposure on the circadian variation of glucose. It has been shown in both lab mice and captive zebra finches that exposure to ALAN can alter feeding behaviour and cause changes to activity-rest cycles, leading to them feeding and being active at unnatural times (Fonken et al., 2010; Batra et al., 2019). For example, diurnal captive zebra finches exposed to dim ALAN, were shown to forage at night, which resulted in elevated nighttime glucose levels, similar to our findings. (Batra et al., 2019).

When comparing glucose levels across treatment groups at the start and end of the experiment, males in the DARK group showed a reduction in glucose levels over time which was not present in females. Individuals exposed to FLAN also experienced a decrease, but it was less pronounced. These differences could be caused by sex-specific digestive characteristics, as seen in Palestine sunbirds *(Cinnyris Osea),* where males had a longer digestion time and lower digestive capacity than females (Markman et al., 2006). Studies investigating sex differences in glucose levels in birds show mixed results (Remage-Healey and Romero, 2000; Tomasek et al., 2019; Pouadjeu et al., 2023). One study found that glucose levels were higher in female free-living passerine birds (Tomasek et al., 2019), while other studies found no evidence of sex differences in glucose levels in either captive starlings *(Sturnus vulgaris)* or free-living tropical passerine birds (Remage-Healey and Romero, 2000; Pouadjeu et al., 2023). Variations in glucose levels over time may depend on many factors including sex, life stage and environment.

The findings in our current study suggest that comparing two static measurements of glucose over time provide little insights into the impact of ALAN on glucose levels. Instead, ALAN effects are only revealed when the circadian variation in glucose concentration is considered. There could be serious implications of circadian misalignment in blood glucose levels, as studies have shown that it can lead to metabolic abnormalities such as obesity, type 2 diabetes mellitus and cardiovascular disease (Kumar Jha et al., 2015; Mason et al., 2020). While birds are known to exhibit a high tolerance for elevated glucose levels, there is evidence to suggest that disruptions to glucose homeostasis can still result in adverse fitness outcomes. For example, baseline glucose levels in adult zebra finches have been shown to be a consistent individual trait that is negatively associated with survival (Montoya et al., 2022). Similarly, chronically high blood glucose levels in collared flycatchers *(Ficedula albicollis)* have been linked to a higher rate of mortality (Récapet et al., 2016). Additionally, in a study on wild great tits, blood glucose levels were associated with fledging success in nestlings (Kaliński et al., 2015). This highlights that misalignment of glucose could have downstream consequences for fitness in wild populations potentially impacting survival and reproductive success.

### 4.2 The impact of ALAN exposure on telomere shortening over time

We observed a trend suggesting that exposure to FLAN may accelerate telomere loss over the course of the experiment. However, this effect was only statistically significant in an exploratory model where the PLAN treatment group was excluded. The full model including all treatment groups did not reveal a significant effect of treatment. While the reduced model suggests a possible effect of FLAN, we acknowledge that this finding should be interpreted with caution, given the non-significance of the global model. Limited statistical power due to small sample sizes, may have masked potential treatment effects in the full model. There are limited studies that have investigated the effects of ALAN exposure on telomere loss and the studies that have been done show mixed results. Several studies report decreased telomere length over time (Liang et al., 2011; Vardi-Naim et al., 2022) while others show no effect (Ouyang et al., 2017; Grunst et al., 2019). For example, several studies show that when exposed to ALAN there is no impact on telomere loss in both nestling and adult songbirds (Ouyang et al., 2017; Grunst et al., 2019). Our findings add nuance to this discussion, suggesting that full ALAN exposure may pose a greater risk than partial ALAN, although more robust sample sizes are needed to confirm these effects.

External stressors have been shown to negatively impact telomere length in various species, for example chronic noise exposure significantly reduced telomere length in nestling house sparrows *(Passer domesticus)* (Meillère et al., 2015). Another study found that great tit nestlings raised in urban habitats had shorter telomeres than those raised in rural habitats (Salmón et al., 2016). These findings suggest that other urban stressors may influence telomere loss to a greater extent than ALAN. The relationship we detected may be due to the study’s duration, as previous studies that showed no effect of ALAN on telomere length were based in the field for a maximum of 15 days (Ouyang et al., 2017; Grunst et al., 2019). A 6-month study on captive zebra finches also found no relationship between ALAN and telomere loss but this study used a lower light intensity (0.3 lux) compared to ours (Alaasam et al., 2024). An acceleration in telomere shortening could lead to negative consequences for the fitness and survival of the organism. Studies looking at bird species have shown that telomere shortening is correlated with increased mortality (Salomons et al., 2009; Barrett et al., 2013; Boonekamp et al., 2014).

### 4.3 The impact of ALAN exposure on oxidative stress over time

We found no evidence that ALAN affected oxidative stress levels over time in captive zebra finches. In females, MDA levels increased in the PLAN group but decreased in both the FLAN and DARK groups over the course of the experiment. For males, only the FLAN group showed an increase in their MDA levels, however these results were not significant. There was also no significant effect of ALAN on OXY levels, although the FLAN group exhibited an increase in OXY over the experiment when compared to the PLAN and DARK groups. These findings agree with studies that reported no effect of ALAN on oxidative stress levels (Raap et al., 2016; Dimovski and Robert, 2018; Czarnecka et al., 2022). However, other studies suggest potential impacts of ALAN. For example, male rats exposed to constant light showed an increase in oxidative stress, marked by elevated MDA levels and reduced antioxidant enzyme activity (Cruz et al., 2003). Several oxidative stress biomarkers have also been shown to be under circadian control in the rat hippocampus, for example rhythmic lipoperoxidation, catalase and GPX expression and activity (Navigatore Fonzo et al., 2009). Catalase and MDA have also been shown to exhibit circadian rhythms in broiler chickens (Ayo et al., 2018).

We observed sex differences in MDA levels between the start and end of the experiment. Males and females often differ in energy investment towards reproduction and traits that are under natural and sexual selection (Pape Møller and Thornhill, 1998; Kokko and Jennions, 2008), which may influence how they respond to environmental stressors (Owens and Hartley, 1998). Some studies have found sex-based differences in oxidative stress levels in response to stress (Kamper et al., 2009; Costantini, 2018; Gomes et al., 2019). For example, in two species of Neotropical manakins, males experienced higher levels of oxidative damage than females when under environmental stress. However, other studies report no sex differences in oxidative stress levels (Costantini et al., 2006; 2007). This does highlight however that it may be important to consider sex differences when measuring oxidative stress levels in response to an environmental stressor.

In addition to an increase in MDA levels in FLAN males, birds in the FLAN group experienced accelerated telomere shortening. Previous studies suggest that increased oxidative stress can increase rate of telomere attrition through DNA damage in the telomere sequence (Kawanishi and Oikawa, 2004; Coluzzi et al., 2014; Reichert and Stier, 2017; Ahmed and Lingner, 2018). However, our analysis did not find any evidence that linked MDA levels to telomere shortening.

### 4.4 Implications for ALAN mitigation

Our study shows that the birds exposed to PLAN did not experience the same physiological changes than the birds exposed to FLAN. Partial night lighting is an emerging mitigation strategy to reduce the ecological impact of ALAN, which involves limiting the duration of illumination in urban environments (Gaston et al., 2012; Jägerbrand and Bouroussis, 2021). This approach therefore reduces the time organisms are exposed to ALAN by switching off streetlights during periods of low human activity or at ecologically critical times, such as during migration or breeding. For example, in Devon, UK many rural towns implement a lighting curfew between 00:30 and 05:30, which has been shown to significantly lower energy consumption (Gaston et al., 2012). Similarly, in Cilaos, Reunion Island, streetlights are turned off during the fledging period of the Barau’s petrel *(Pterodroma baraui)* to reduce light-induced mortality (Rodriguez et al., 2015). Strategies such as partial night lighting are desirable as the complete removal of ALAN is impractical due to concerns about human safety and societal needs, however there are still concerns regarding crime and traffic safety during unlit hours (Jägerbrand and Bouroussis, 2021). Despite these challenges, this strategy holds promises which we display in the present study. However, evidence supporting the effectiveness of partial light at night strategies remain limited, particularly when considering physiological outcomes. Therefore, more studies are needed to reevaluate the biological efficacy of this approach before widespread implementation can take place in key biodiversity areas.

### 4.5 Limitations and future directions

One of the main limitations in the current study was that we were constrained by a relatively small sample size, therefore any interactions showing weak or marginal significance should be interpreted with caution. Further studies with larger sample sizes are needed to confirm the patterns identified in this study which would further strengthen our findings. Another limitation of the present study is the absence of data on activity levels and feeding behaviour, which could have provided deeper insight into the physiological changes we observed in the zebra finches. Future research could incorporate the use of RFID tags to monitor individual feeding times by pairing them with specially designed feeders that log both the bird identity and timestamp of each visit. Alternatively, continuous video recording using cameras installed in each cage could capture patterns of activity and feeding behaviour during both the day and night. These additions would be particularly valuable given the observed variation in glucose levels observed in the birds exposed to FLAN, which is likely to have resulted from altered nocturnal activity and feeding. Understanding these mechanisms would allow us to clarify the ways in which ALAN influences metabolism.

In the present study we were only able to assess two biomarkers of oxidative stress due to sample size constraints. Given the complexity of the oxidative stress system, it is optimal to evaluate multiple biomarkers that encompass both oxidative damage and antioxidant defence across different tissues to accurately capture the individual’s oxidative status (Beaulieu and Costantini, 2014). We also emphasis the value of incorporating oxidative stress markers that are more directly linked to DNA and telomere damage such as 8-hydroxy-2′-deoxyguanosine (8-OHdG) and 8-oxo-guanine (8-oxoG) in future studies. These biomarkers could offer mechanistic insights into how ALAN exposure may contribute to telomere attrition (Prabhulkar and Li, 2010; Coluzzi et al., 2014; Domínguez-de-Barros et al., 2024).

## 5 Conclusions

Our results highlight that exposure to full ALAN impacts the circadian pattern of glucose in captive zebra finches, causing delayed glucose peaks compared to controls, which could lead to metabolic abnormalities and reduced fitness. We also observed a potential acceleration in telomere shortening under full ALAN exposure, however this effect was not supported by the full model. As such, this finding should be interpreted with caution and warrants further investigation. In contrast, partial ALAN did not produce these effects, indicating that it may be a biologically safer alternative. A key limitation of this study was the small sample size in each treatment group, which may have limited the detection of significant effects in the full global models. Nonetheless, our findings provide preliminary support for partial night lighting as a mitigation strategy in urban environments. This could include switching artificial lights off after midnight to reduce ALAN’s impact on urban wildlife. In addition to ecological benefits, this strategy may offer economic advantages through reduced energy costs and contribute to reducing emissions. Our study therefore contributes to the growing evidence base for managing light pollution more sustainably.

## Data Availability

The datasets presented in this study can be found in online repositories. The names of the repository/repositories and accession number(s) can be found below: Zenodo at [https://doi.org/10.5281/zenodo.15011283].
