# Correction: Partial night lighting may reduce the physiological impact of artificial light at night on captive zebra finches

**DOI:** 10.3389/fphys.2025.1644617

**Published:** 2025-08-29

**Authors:** Rachel R. Reid, Neal Dawson, Eleanor Duncan, Robert Gillespie, Christopher Mitchell, Claire J. Branston, Pablo Capilla-Lasheras, Jelle Boonekamp, Davide M. Dominoni

**Affiliations:** ^1^ School of Biodiversity, One Health and Veterinary Medicine, Graham Kerr Building, University of Glasgow, Glasgow, United Kingdom; ^2^ Environment and Sustainability Institute, University of Exeter, Cornwall, United Kingdom; ^3^ School of Health and Life Sciences, University of the West of Scotland, Glasgow, United Kingdom; ^4^ Swiss Ornithological Institute (Vogelwarte), Sempach, Switzerland; ^5^ Doñana Biological Station, Spanish National Research Council (EBD-CSIC), Sevilla, Spain

**Keywords:** avian health, avian physiology, artificial light, light pollution, urban ecology

In the published article, there was an error. [Inter-plate and intra-plate CV were labelled incorrectly for relative telomere length].

A correction has been made to **[Methods]**, [*2.5 Measuring relative telomere length*], [Paragraph 4]. This sentence previously stated:

“[Mean inter- and intra-plate of Ct values were 2.14% and 2.97% for telomere reactions and 1.02% and 1.10% for the RAG-1 reactions.]”

The corrected sentence appears below:

“[Mean intra- and inter-plate of Ct values were 2.14% and 2.97% for telomere reactions and 1.02% and 1.10% for the RAG-1 reactions.]”

In the published article, there was an error. [Inter-plate and intra-plate CV were labelled incorrectly for antioxidant capacity of plasma].

A correction has been made to **[Methods]**, [*2.7 Measuring antioxidant capacity of plasma*], [Paragraph 2]. This sentence previously stated:

“[The between plate repeatability was R 2 = 0.57 (N = 20). The inter-plate CV calculated using these same 20 samples was 9.35% and the intra-plate CV was 10.97%.]”

The corrected sentence appears below:

“[The between plate repeatability was R 2 = 0.57 (N = 20). The intra-plate CV calculated using these same 20 samples was 9.35% and the inter-plate CV was 10.97%.]”

The original article has been updated.

