# Supplementary material for: Partial night lighting may reduce the physiological impact of artificial light at night on captive zebra finches
Source: Front Physiol. 2025 May 30;16:1592407. doi: 10.3389/fphys.2025.1592407 (PMC12162287; doi:10.3389/fphys.2025.1592407)
Supplement: Supplementary file 1 [file DataSheet1.docx]

Supplementary materials: Partial night lighting has the potential to reduce the physiological impact of artificial light at night in captive zebra finches.

***Table S1:*** *The table below shows the output of the model testing the effect of fixed effects on the circadian variation of glucose with the values from Drop1 test. The output shows the model estimate, standard error, 95% confidence intervals, degrees of freedom and P values. The fixed effects can be seen in bold, and any factor levels are shown in italics. A SE = Standard Error, CI = Confidence Interval*

| **Fixed effect** | **Estimate** | **SE***^A^* | **95% CI***^A^* | **χ^2^** | **df** | **p-value** |
| --- | --- | --- | --- | --- | --- | --- |
| **Intercept** | 16 | 7.529 | 1.5, 31 |  |  |  |
| **Weight** | -0.06 | 0.277 | -0.61, 0.48 | 0.08 | 1 | 0.774 |
| **Treatment** |  |  |  |  |  |  |
| *DARK* | — | — | — |  |  |  |
| *FLAN* | -0.23 | 2.312 | -4.8, 4.3 |  |  |  |
| *PLAN* | 2.9 | 2.486 | -2.0, 7.7 |  |  |  |
| **Sex** |  |  |  |  |  |  |
| *Female* | — | — | — |  |  |  |
| *Male* | 2.0 | 2.505 | -2.9, 6.9 |  |  |  |
| **Time of day** |  |  |  |  |  |  |
| *1am* | — | — | — |  |  |  |
| *1pm* | -3.5 | 2.232 | -7.9, 0.84 |  |  |  |
| *6am* | -1.4 | 2.509 | -6.3, 3.5 |  |  |  |
| *8pm* | 3.5 | 2.611 | -1.7, 8.6 |  |  |  |
| **Group size** | -1.0 | 0.986 | -2.9, 0.92 | 1.61 | 1 | 0.205 |
| **Treatment * Sex * Time of day** |  |  |  | 31.90 | 17 | **0.015** |
| *DARK * Female * 1am* | 2.5 | 4.847 | -7.0, 12 |  |  |  |
| *FLAN * Female * 1am* | 8.1 | 4.849 | -1.4, 18 |  |  |  |
| *PLAN * Female * 1am* | 3.2 | 3.504 | -3.6, 10 |  |  |  |
| *DARK * Male * 1am* | -1.4 | 3.961 | -9.2, 6.4 |  |  |  |
| *FLAN * Male * 1am* | 4.9 | 3.516 | -2.0, 12 |  |  |  |
| *DARK * Female * 1pm* | 4.7 | 4.842 | -4.8, 14 |  |  |  |
| *FLAN * Female * 1pm* | 8.2 | 4.973 | -1.5, 18 |  |  |  |
| *PLAN * Female * 1pm* | 3.5 | 3.582 | -3.5, 11 |  |  |  |
| *DARK * Male * 1pm* | -0.16 | 3.964 | -7.9, 7.6 |  |  |  |
| *FLAN * Male * 1pm* | 6.6 | 3.508 | -0.27, 13 |  |  |  |
| *DARK * Female * 6am* | 0.08 | 4.273 | -8.3, 8.5 |  |  |  |
| *FLAN * Female * 6am* | 3.5 | 4.071 | -4.4, 12 |  |  |  |
| *PLAN * Female * 6am* | -1.6 | 2.527 | -6.6, 3.3 |  |  |  |
| *DARK * Male * 6am* | 5.1 | 2.526 | 0.11, 10 |  |  |  |
| *FLAN * Male * 6am* | 2.9 | 2.475 | -2.0, 7.7 |  |  |  |
| *DARK * Female * 8pm* | 3.0 | 3.396 | -3.6, 9.7 |  |  |  |
| *FLAN * Female * 8pm* | 2.8 | 3.146 | -3.4, 9.0 |  |  |  |
| *^A^* SE = Standard Error, CI = Confidence Interval | | | | | | |

***Table S2:*** *The table below shows the output of the post hoc analysis for pairwise comparison between the treatment groups at each sex level and time of day level when investigating the impact of treatment group, sex and time of day on the circadian variation of glucose.*

| Contrast | Sex | Timepoint | Est | SE | Cl.lower | Cl.upper | P value |
| --- | --- | --- | --- | --- | --- | --- | --- |
| DARK-FLAN | Female | 1pm | -3.336 | 2.62 | -9.68 | 3.010 | 0.417 |
| DARK-PLAN | Female | 1pm | -1.694 | 2.50 | -7.76 | 4.367 | 0.777 |
| FLAN-PLAN | Female | 1pm | 1.642 | 2.81 | -5.16 | 8.448 | 0.829 |
| **DARK-FLAN** | **Male** | **1pm** | **-6.535** | **2.59** | **-12.85** | **-0.224** | **0.041** |
| DARK-PLAN | Male | 1pm | -3.013 | 2.78 | -9.76 | 3.738 | 0.528 |
| FLAN-PLAN | Male | 1pm | 3.522 | 2.56 | -2.70 | 9.748 | 0.363 |
| DARK-FLAN | Female | 6am | -3.215 | 2.46 | -9.21 | 2.782 | 0.400 |
| DARK-PLAN | Female | 6am | -1.164 | 2.32 | -6.79 | 4.461 | 0.870 |
| FLAN-PLAN | Female | 6am | 2.051 | 2.43 | -3.87 | 7.967 | 0.678 |
| DARK-PLAN | Male | 6am | 2.401 | 2.18 | -2.90 | 7.700 | 0.519 |
| DARK-PLAN | Male | 6am | 2.208 | 2.37 | -3.56 | 7.980 | 0.623 |
| FLAN-PLAN | Male | 6am | -0.193 | 2.23 | -5.63 | 5.241 | 0.996 |
| **DARK-FLAN** | **Female** | **1am** | **-5.364** | **2.35** | **-11.08** | **0.347** | **0.07** |
| DARK-PLAN | Female | 1am | -3.572 | 2.37 | -9.35 | 2.210 | 0.299 |
| FLAN-PLAN | Female | 1am | 1.792 | 2.44 | -4.14 | 7.726 | 0.744 |
| **DARK-FLAN** | **Male** | **1am** | **-6.060** | **2.59** | **-12.37** | **0.251** | **0.062** |
| DARK-PLAN | Male | 1am | -4.250 | 2.77 | -10.99 | 2.492 | 0.286 |
| FLAN-PLAN | Male | 1am | 1.810 | 2.58 | -4.45 | 8.075 | 0.763 |
| DARK-FLAN | Female | 8pm | 0.469 | 2.44 | -5.48 | 6.419 | 0.98 |
| DARK-PLAN | Female | 8pm | 0.183 | 2.31 | -5.44 | 5.801 | 0.997 |
| FLAN-PLAN | Female | 8pm | -0.287 | 2.42 | -6.19 | 5.620 | 0.992 |
| DARK-FLAN | Male | 8pm | 0.234 | 2.32 | -5.39 | 5.857 | 0.994 |
| DARK-PLAN | Male | 8pm | -2.854 | 2.49 | -8.91 | 3.204 | 0.492 |
| FLAN-PLAN | Male | 8pm | -3.088 | 2.50 | -9.15 | 2.970 | 0.438 |

***Table S3:*** *The table below shows the output of the model testing the effect of fixed effects on the glucose levels at the start and end of the experiment with the values from Drop1 test. The output shows the model estimate, standard error, 95% confidence intervals, degrees of freedom and P values. The fixed effects can be seen in bold, and any factor levels are shown in italics. A SE = Standard Error, CI = Confidence Interval*

| **Fixed effect** | **Estimate** | **SE***^A^* | **95% CI***^A^* | **χ^2^** | **df** | **p-value** |
| --- | --- | --- | --- | --- | --- | --- |
| **Intercept** | 14 | 4.850 | 4.8, 24 |  |  |  |
| **Weight** | -0.04 | 0.261 | -0.55, 0.47 | 0.00 | 1 | 0.990 |
| **Treatment** |  |  |  | 1.68 | 2 | 0.431 |
| *DARK* | — | — | — |  |  |  |
| *FLAN* | 1.4 | 1.116 | -0.80, 3.6 |  |  |  |
| *PLAN* | 0.76 | 1.012 | -1.2, 2.7 |  |  |  |
| **Sex** |  |  |  | 0.00 | 1 | 0.952 |
| *Female* | — | — | — |  |  |  |
| *Male* | 0.04 | 0.915 | -1.8, 1.8 |  |  |  |
| **Experiment timepoint** |  |  |  | 0.07 | 1 | 0.797 |
| *End* | — | — | — |  |  |  |
| *Start* | -0.14 | 0.632 | -1.4, 1.1 |  |  |  |
| **Group size** | 0.35 | 0.585 | -0.80, 1.5 | 0.45 | 1 | 0.503 |
| *^A^* SE = Standard Error, CI = Confidence Interval | | | | | | |

***Table S4:*** *The table below shows the output of the model testing the effect of fixed effects on relative telomere length with the values from Drop1 test. The output shows the model estimate, standard error, 95% confidence intervals, degrees of freedom and P values. The fixed effects can be seen in bold, and any factor levels are shown in italics. A SE = Standard Error, CI = Confidence Interval*

| **Fixed effect** | **Estimate** | **SE***^A^* | **95% CI***^A^* | **χ^2^** | **df** | **p-value** |
| --- | --- | --- | --- | --- | --- | --- |
| **Intercept** | 1.4 | 0.868 | -0.35, 3.1 |  |  |  |
| **Weight** | -0.01 | 0.043 | -0.10, 0.07 | 0.12 | 1 | 0.734 |
| **Treatment** |  |  |  | 1.28 | 2 | 0.528 |
| *DARK* | — | — | — |  |  |  |
| *FLAN* | -0.02 | 0.261 | -0.54, 0.49 |  |  |  |
| *PLAN* | 0.21 | 0.253 | -0.28, 0.71 |  |  |  |
| **Sex** |  |  |  | 0.34 | 1 | 0.560 |
| *F* | — | — | — |  |  |  |
| *M* | 0.12 | 0.228 | -0.32, 0.57 |  |  |  |
| **Experiment timepoint** |  |  |  | 0.81 | 1 | 0.367 |
| *End* | — | — | — |  |  |  |
| *Start* | 0.06 | 0.066 | -0.07, 0.19 |  |  |  |
| **Group size** | -0.03 | 0.145 | -0.31, 0.26 | 0.04 | 1 | 0.844 |
| *^A^* SE = Standard Error, CI = Confidence Interval | | | | | | |

***Table S5:*** *The table below shows the output of the model testing the effect of fixed effects on MDA levels with the values from Drop1 test. The output shows the model estimate, standard error, 95% confidence intervals, degrees of freedom and P values. The fixed effects can be seen in bold, and any factor levels are shown in italics. A SE = Standard Error, CI = Confidence Interval*

| **Fixed effect** | **Estimate** | **SE***^A^* | **95% CI***^A^* | **χ^2^** | **df** | **p-value** |
| --- | --- | --- | --- | --- | --- | --- |
| **Intercept** | -0.89 | 4.024 | -8.8, 7.0 |  |  |  |
| **Weight** | 0.39 | 0.246 | -0.09, 0.87 | 2.72 | 1 | 0.099 |
| **Treatment** |  |  |  | 0.23 | 2 | 0.891 |
| *DARK* | — | — | — |  |  |  |
| *FLAN* | 0.32 | 0.949 | -1.5, 2.2 |  |  |  |
| *PLAN* | -0.11 | 0.963 | -2.0, 1.8 |  |  |  |
| **Sex** |  |  |  | 5.49 | 1 | **0.019** |
| *Female* | — | — | — |  |  |  |
| *Male* | -1.7 | 0.743 | -3.2, -0.24 |  |  |  |
| **Experiment timepoint** |  |  |  | 0.93 | 1 | 0.335 |
| *End* | — | — | — |  |  |  |
| *Start* | 0.68 | 0.734 | -0.76, 2.1 |  |  |  |
| *^A^* SE = Standard Error, CI = Confidence Interval | | | | | | |


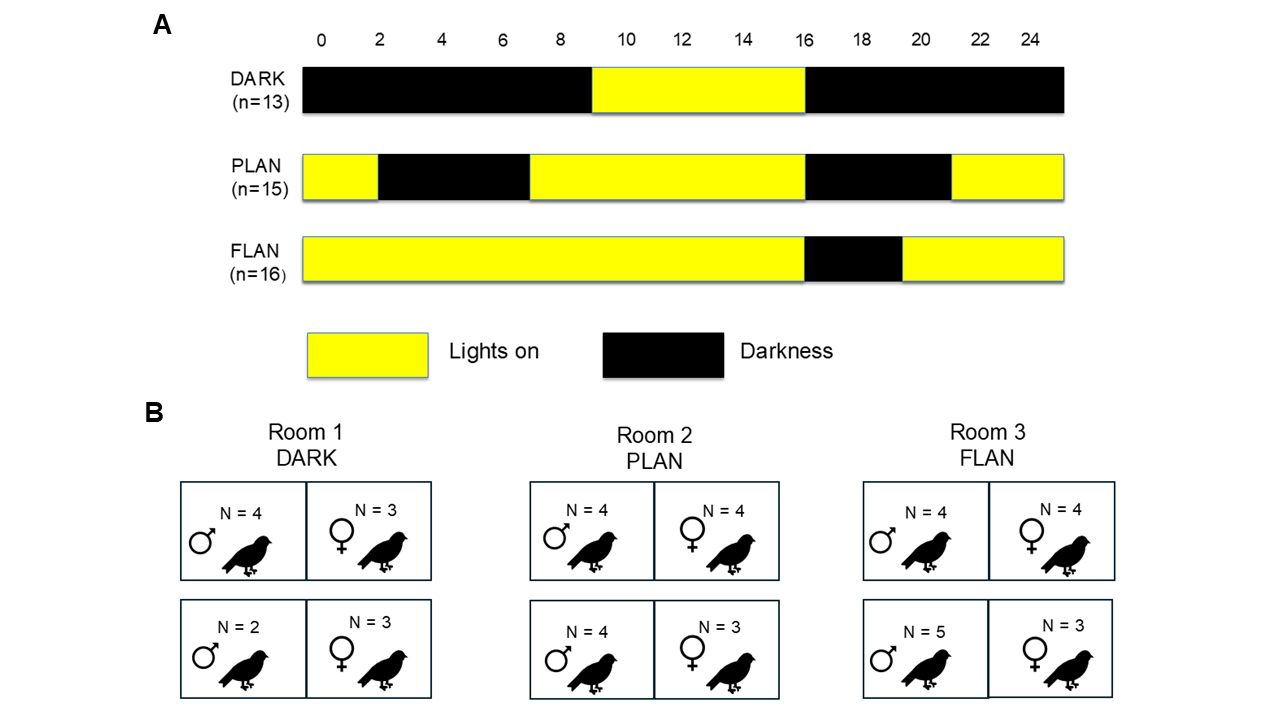


***Figure S1:*** *Experimental setup showing the light/dark schedule that each treatment group was exposed to with time (24-hour format) shown on the y axis. The full sample sizes for each treatment group are also shown (Panel A). Panel B shows the bird room composition, split by treatment group and sex, along with the corresponding sample sizes.*


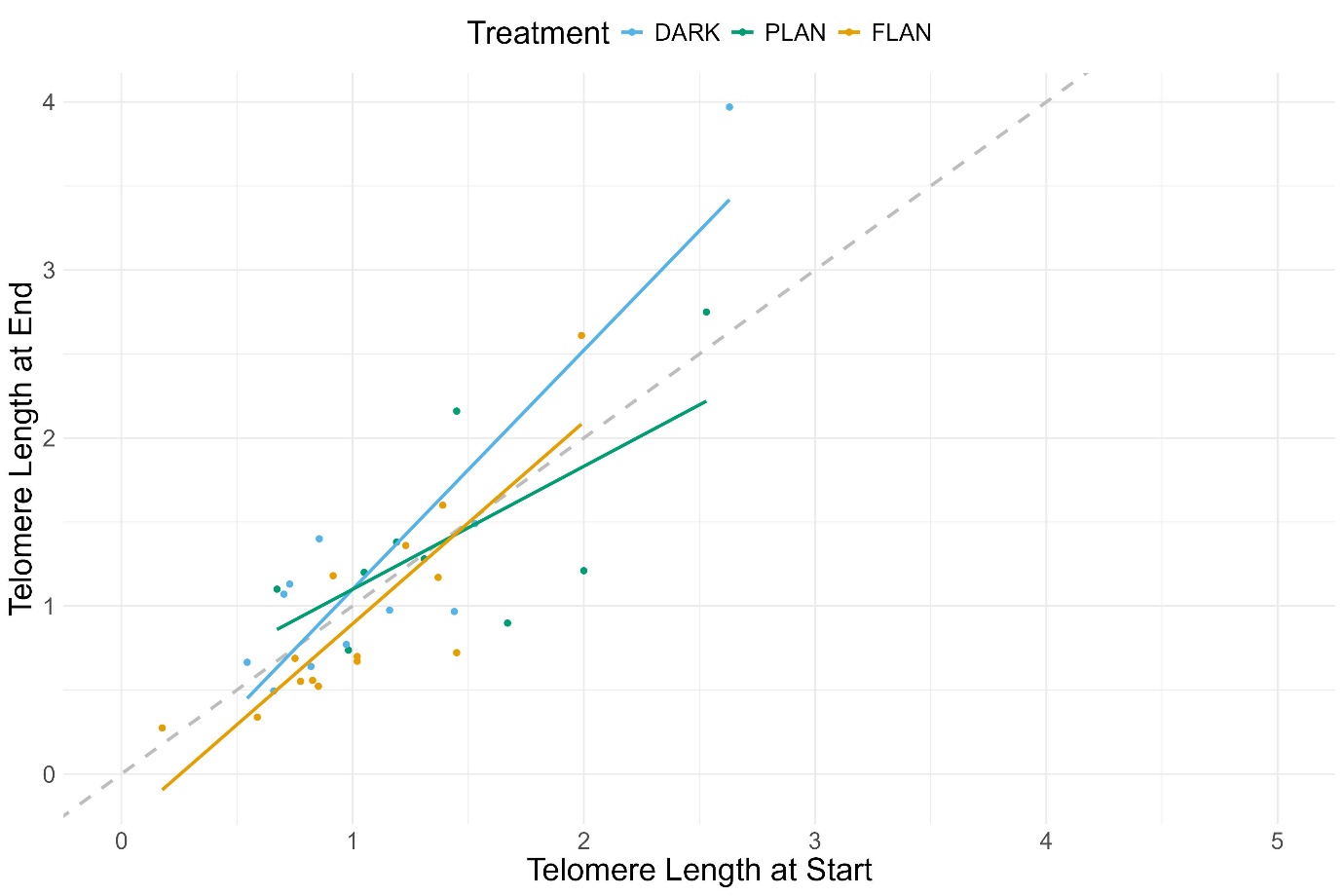


***Figure S2:*** *This figure shows Telomere length for each individual at the start of the experiment plotted against telomere length at the end of the experiment. The grey dotted line plotted through zero represents y=x. The different coloured regression lines represent the different treatment groups (DARK, PLAN, FLAN). Telomere loss is shown by the amount of data points that lie beneath the y=x line.*

***Table S6:*** *The table below shows the output of the model testing the effect of fixed effects on OXY levels with the values from Drop1 test. The output shows the model estimate, standard error, 95% confidence intervals, degrees of freedom and P values. The fixed effects can be seen in bold, and any factor levels are shown in italics. A SE = Standard Error, CI = Confidence Interval*

| **Fixed effect** | **Estimate** | **SE***^A^* | **95% CI***^A^* | **χ^2^** | **df** | **p-value** |
| --- | --- | --- | --- | --- | --- | --- |
| **Intercept** | 37 | 92.037 | -144, 217 |  |  |  |
| **Weight** | 13 | 4.917 | 3.4, 23 | 6.99 | 1 | **0.008** |
| **Treatment** |  |  |  | 1.08 | 2 | 0.583 |
| *DARK* | — | — | — |  |  |  |
| *FLAN* | -13 | 21.700 | -56, 29 |  |  |  |
| *PLAN* | -20 | 20.499 | -60, 21 |  |  |  |
| **Sex** |  |  |  | 0.10 | 1 | 0.746 |
| *Female* | — | — | — |  |  |  |
| *Male* | -5.2 | 18.499 | -41, 31 |  |  |  |
| **Experiment timepoint** |  |  |  | 0.71 | 1 | 0.400 |
| *End* | — | — | — |  |  |  |
| *Start* | -14 | 14.391 | -42, 14 |  |  |  |
| **Group_size** | 3.8 | 11.754 | -19, 27 | 0.15 | 1 | 0.694 |
| *^A^* SE = Standard Error, CI = Confidence Interval | | | | | | |
